# More than insulator: multiple roles of CTCF at the *H19-Igf2* imprinted domain

**DOI:** 10.3389/fgene.2012.00214

**Published:** 2012-10-15

**Authors:** Purnima Singh, Dong-Hoon Lee, Piroska E. Szabó

**Affiliations:** Department of Molecular and Cellular Biology, Beckman Research InstituteDuarte, CA, USA

**Keywords:** CTCF chromatin, imprinting, H19, Igf2, insulators, methylation, Zfp57, Trim28

## Abstract

CTCF (CCCTC-binding factor)-mediated insulation at the *H19-Insulin-like growth factor* 2* (Igf2)* imprinted domain is a classic example for imprinted gene regulation. DNA methylation difference in the imprinting control region (ICR) is inherited from the gametes and subsequently determines parental allele-specific enhancer blocking and imprinted expression in the soma. Recent genetic studies showed that proper monoallelic enhancer blocking at the *H19-Igf2* ICR is critical for development. Strict biallelic insulation at this locus causes perinatal lethality, whereas leaky biallelic insulation results in smaller size but no lethality. Apart from enhancer blocking, CTCF is also the master organizer of chromatin composition in the maternal allele along this imprinted domain, affecting not only histone tail covalent modifications but also those in the histone core. Additionally, CTCF binding in the soma protects the maternal allele from *de novo* DNA methylation. CTCF binding is not involved in the establishment of the gametic marks at the ICR, but it slightly delays *de novo* methylation in the maternally inherited ICR allele in prospermatogonia. This review focuses on the developmental and epigenetic consequences of CTCF binding at the *H19-Igf2* ICR.

CTCF (also known as CCCTC-binding factor) is a major organizer of the vertebrate genome and is essential for development (Moore et al., 2012). It is a versatile protein that regulates gene expression by binding to DNA via its multiple zinc fingers (Filippova, 2008; Ohlsson et al., 2010; Herold et al., 2012). CTCF plays roles in transcriptional activation and repression, insulation by enhancer blocking or chromosome barrier formation and organization of higher order chromatin by chromosomal looping and nuclear tethering (Phillips and Corces, 2009; Weth and Renkawitz, 2011; Barkess and West, 2012; Ghirlando et al., 2012). CTCF has been implicated in such diverse biological phenomena as genomic imprinting, X chromosome inactivation (Spencer et al., 2011), alternative splicing (Shukla et al., 2011), microsatellite instability (Libby et al., 2008), and V(D)J recombination (Guo et al., 2011). Several methodologies have been utilized for testing CTCF's function, including *in vitro* and cell culture assays, depletion or ablation of CTCF and its interactive partners, and deleting CTCF sites from episomal vectors, integrated transgenes or endogenous loci. The most direct functional test is to specifically inactivate the CTCF binding site(s) at an endogenous locus by point mutations. To date almost no such genetic studies exist in the latter category. One notable exception is the mouse *H19-Igf2* imprinted domain, which has been extensively studied in the past decade by several independent groups including ours. Precise point mutations have been made that inactivated the CTCF binding sites in the imprinting control region (ICR). In this review we will focus on some of the colorful roles that CTCF plays at the *H19-Igf2* imprinted locus. We will review that CTCF-mediated insulation controls reciprocal parental allele-specific expression of these two imprinted genes, emphasizing that correct monoallelic enhancer blocking at this locus is critical for normal fetal development. We will also summarize the roles CTCF plays in maintaining the epigenetic features of the maternal allele in the soma and, to some extent, in primordial germ cells (PGCs).

## Parental allele-specific enhancer insulation at the *H19-Igf2* imprinted domain

**Figure 1:**
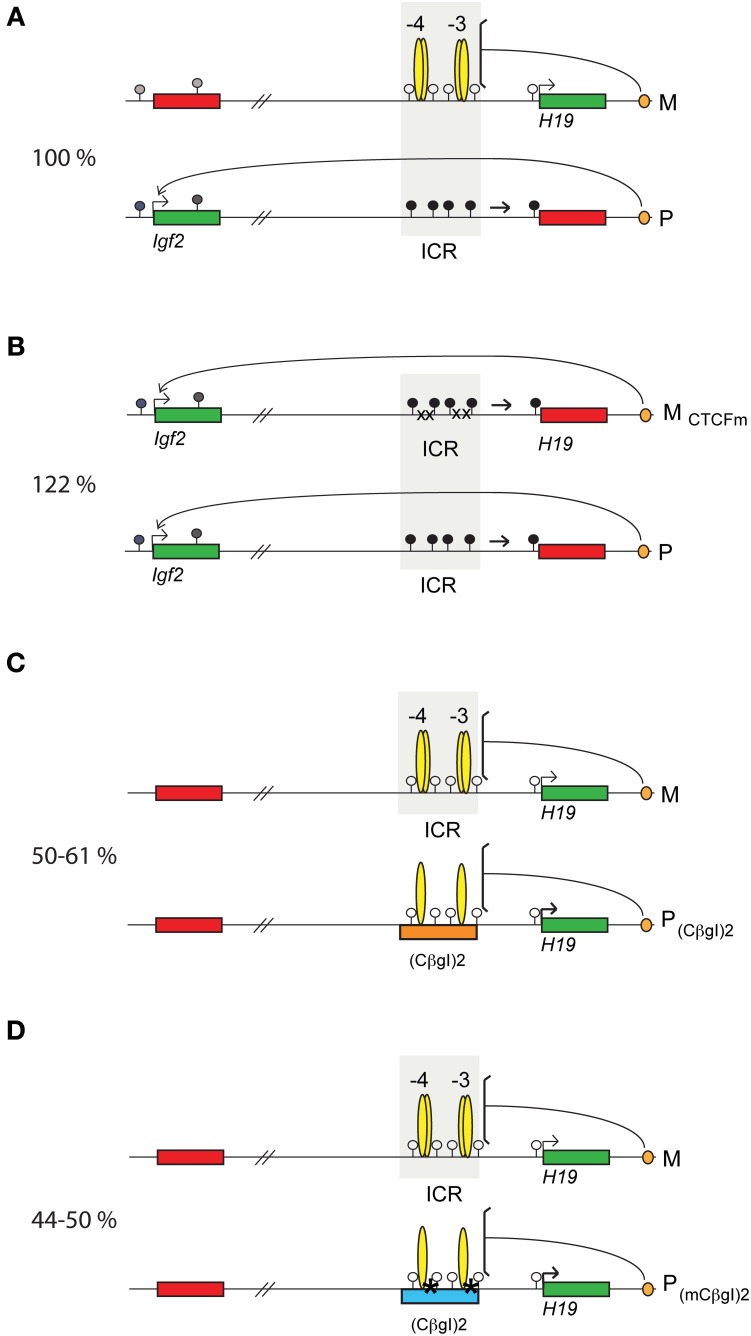
**Parental allele-specific enhancer insulation at the *H19-Igf2* imprinted domain. (A)** Imprinted insulation at the *H19/Igf2* imprinted domain by CTCF binding in the ICR based on publications referenced in the text. Maternal chromosome (M): unmethylated (white lollipops) ICR (shaded area) is inherited from the oocyte. CTCF (yellow ovals) imparts insulator activity (bracket) between the *Igf2* promoters and the shared, downstream enhancers (orange oval). Initiation of *H19* expression depends on an unmethylated ICR during embryogenesis. Paternal chromosome (P): methylated (black lollipops) ICR is inherited from the sperm, CTCF cannot bind, hence ICR has no insulator activity thus, the *Igf2* promoters and enhancers can interact. During early postimplantation development, the *H19* promoter is inactivated by an ICR-dependent mechanism (horizontal arrow). **(B)** CTCF binding site mutations in the maternal ICR allele disrupt imprinted expression (Szabó et al., 2004). CTCF no longer binds in the mutant maternal chromosome (M_CTCFm_), thus, the enhancers can access the *Igf2* promoter in both alleles. The mutant ICR is methylated and inactivates the *H19* promoter. **(C)** Non-imprinted insulation at the *H19*/*Igf2* locus by the chicken β-globin insulator duplex (ChβGI)_2_ (orange rectangle) (Szabó et al., 2002). The (ChβGI)_2_ is unmethylated and insulates the *Igf2* promoter from the shared enhancers when substituted for the ICR and transmitted maternally (not shown) or paternally (P), with 10% *Igf2* activity remaining. *H19* is overactivated 1.5-fold by the (ChβGI)_2_ sequences in the paternal allele (bold arrow). **(D)** Biallelic insulation by the mutant chicken β-globin insulator duplex (mChβGI)_2_ (turquoise rectangle) carrying mutations for boundary factor binding sites (stars) (Lee et al., 2010). Insulation is complete, with no detectable remaining *Igf2* expression. Relative fetus size for each genotype is shown to the left. Active genes and silent genes are depicted with green and red rectangles, respectively.

CTCF-mediated insulation is a classic example for the regulation of genomic imprinting. Imprinted genes exhibit parental allele-specific expression (Ferguson-Smith, 2011; Abramowitz and Bartolomei, 2012). *Insulin-like growth factor* 2 (*Igf2*), and *H19* are neighboring genes, located on distal chromosome 7 in the mouse and expressed from the paternally or maternally inherited chromosome, respectively. Igf2 protein is important for promoting fetal and placental growth (DeChiara et al., 1990; Constancia et al., 2002) whereas the *H19* non-coding RNA moderates growth in the normal fetus (Gabory et al., 2009), puts the brake on the growth of the term placenta via its microRNA (Keniry et al., 2012) and also functions as a tumor suppressor (Yoshimizu et al., 2008). Both genes respond to the same endodermal enhancers that are distal to *H19* (Leighton et al., 1995) (Figure 1A). Between these two genes lies a 2.4 kb long differentially methylated region (DMR) that is required for the monoallelic expression of both the *H19* and *Igf2* genes, and therefore is called an ICR. Its deletion from the maternal allele results in biallelic *Igf2* expression and from the paternal allele in biallelic *H19* expression. Methylation of this DMR is exclusive to the paternally inherited chromosome and originates from the sperm (Tremblay et al., 1995, 1997; Thorvaldsen et al., 1998). *Igf2* expression is also regulated by two additional paternally methylated DMRs. *Igf2* DMR1, upstream of the *Igf2* gene functions as a mesodermal silencer in the maternal allele (Constancia et al., 2000) while DMR2, in the sixth exon, functions as an enhancer in the paternal allele (Murrell et al., 2001).

To shed light on how the ICR regulates reciprocal expression of *Ig2* and *H19*, we used *in vivo* DNAseI, DMS footprinting and UV photofootprinting analysis of mouse embryo fibroblasts (MEFs) carrying maternal or paternal duplication of distal Chromosome 7 and discovered strong footprints at four consensus CTCF binding sites in the unmethylated maternal ICR allele but not in the methylated paternal allele. This provided evidence that the CTCF insulator protein blocks communication between the *Igf2* promoters and the shared downstream enhancers in the maternal chromosome (Szabó et al., 2000). At the same time, *in vitro* enhancer blocking, gelshift, episome assays, and *in vivo* ChIP assays confirmed that the *H19-Igf2* ICR acts as an enhancer blocker in the unmethylated maternal allele and CTCF binding is inhibited in the paternal ICR allele by DNA methylation, allowing *Igf2* promoter access to the enhancers (Bell and Felsenfeld, 2000; Hark et al., 2000; Kanduri et al., 2000). To verify the enhancer blocker role of CTCF at this locus *in vivo*, CTCF-site mutations were introduced into the ICR allele in the mouse. Maternal transmission of these mutations resulted in biallelic *Igf2* expression and biallelic *H19* silencing (Figure 1B) (Pant et al., 2003; Schoenherr et al., 2003; Szabó et al., 2004; Han et al., 2008). CTCF has also been reported to be responsible at this locus for asynchronous replication of the two alleles: late replication of the maternal allele depends on CTCF binding (Bergstrom et al., 2007; Guibert et al., 2012). CTCF-dependent enhancer blocking requires cohesins (Rubio et al., 2008; Stedman et al., 2008; Nativio et al., 2009; Yao et al., 2010; Xiao et al., 2011) and involves regulating chromosome loop formation (Murrell, 2011).

Parental allele-specific CTCF binding has been detected recently at additional imprinted domains, at the *Rasgrf1* (Yoon et al., 2005), *Gtl2* (Lin et al., 2011), *Grb10* (Hikichi et al., 2003), *Kcnq1/Kcnq1ot1* (Fitzpatrick et al., 2007), and *Peg13* DMRs (Singh et al., 2011). It will be very interesting to test using genetic analyses whether these CTCF binding sites are required for regulating the allele-specific expression of imprinted transcripts by enhancer blocking.

## Monoallelic insulation at the *H19-Igf2* ICR is essential for normal development

**Figure 2:**
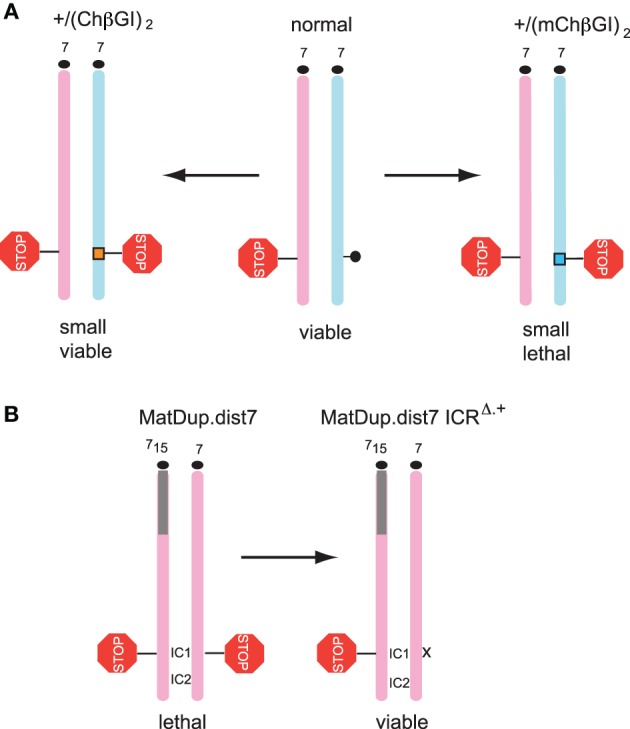
**Biallelic insulation at the ICR is not tolerated in development. (A)** Introducing strict biallelic insulation to the ICR causes lethality. Substituting the paternal chromosome's (light blue) methylated (black lollipop) ICR of normal mice (middle) with the (ChβGI)_2_ (Szabó et al., 2002) (orange box) or the (mChβGI)_2_ (Lee et al., 2010) (turquoise box) has resulted in biallelic insulation (STOP signal). Lethality was observed in the +/(mChβGI)_2_ but not in the +/(ChβGI)_2_ genotype. The +/(mChβGI)_2_ had strict insulation but the +/(ChβGI)_2_ exhibited leaky insulation. **(B)** Maternal (pink) duplication of distal chromosome 7 (MatDup.dist7) fetuses that carry biallelic insulation at the ICR, also called imprinting control center 1 (IC1), have 40% body weight and die. The lethality phenotype is rescued by maternal transmission of one copy of the mutant IC1 (x) that lacks CTCF binding and insulator function (Han et al., 2010). The imprinting control center 2 (IC2) is bi-maternal. Correction of biallelic ICR insulation to monoallelic insulation is sufficient to rescue perinatal lethality of the MatDup.dist7 genotype.

Genetic studies revealed that insulation strength of the *H19-Igf2* ICR has consequences to body size and viability. Insulation was absent at the *H19-Igf2* domain in mice carrying the ICR CTCF site mutations in the maternal chromosome. This resulted in elevated *Igf2* expression and an overgrowth phenotype (Figure 1B). Prenatal fetuses were 122% heavier than their normal siblings (Szabó et al., 2004). We also noticed that adult males that carried the ICR CTCF site mutations became aggressive and fought frequently. Insulation was biallelic at this locus in mice where the ICR was replaced with two copies of the chicken beta globin insulator (ChβGI)_2_ (Figure 1C) (Szabó et al., 2002). This introduced DNA fragment was of similar size to the ICR, had two CTCF binding sites, and also included sufficient number of CpG dinucleotides. The (ChβGI)_2_ functioned as an enhancer blocker in the maternal allele. In the paternal allele, however, it behaved differently from the endogenous ICR. The (ChβGI)_2_ did not attain *de novo* methylation in the male germ line and thus, it was not methylated in the paternally inherited allele in the somatic organs of +/(ChβGI)_2_ fetuses. It consequently allowed biallelic CTCF binding and insulation of the *Igf2* promoters from the shared enhancers. *Igf2* expression was reduced to 10% of normal values and fetus size was reduced to 50–61% of normal littermates. *H19* expression was biallelic. Later a very similar mouse model was generated (Lee et al., 2010) that carried a mutant form of the (mChβGI)_2_ sequences (Figure 1D). CTCF binding sites were retained in the (mChβGI)_2_ but consensus sites for boundary proteins, USF1 (West et al., 2004; Yao et al., 2010) and VEZF1 (Clark et al., 1990; Dickson et al., 2010), were destroyed by point mutations. Although there was a slight, 32%, methylation at these sequences in the male germ line, paternal allele-specific methylation was not maintained in the soma. In +/(mChβGI)_2_ offspring insulation was again biallelic, and even more strict than the insulation in +/(ChβGI)_2_ fetuses. *Igf2* expression was undetectable and fetus size was reduced to 44–50% of normal littermates. Whereas the +/(ChβGI)_2_ mice were viable, a fully penetrant perinatal lethality occurred in the +/(mChβGI)_2_ genotype (Figure 2A). The absence of *Igf2* likely contributed to the lethality phenotype of +/(mChβGI)_2_, but was not the sole cause, because *Igf2* homozygous mutant mice are small but viable (DeChiara et al., 1990). Similar conclusion was reached in the reciprocal experiment (Figure 2B), when perinatal lethality of mice carrying maternal duplication of distal chromosome 7 (MatDup.dist7) was rescued by introducing the CTCF site mutations into one allele of the *H19-Igf2* ICR (also called IC1) (Han et al., 2010). Correcting biallelic insulation of the *H19-Igf2* ICR was sufficient to rescue lethality, even though the duplicated chromosome region of MatDup.dist7 mice also carries the Kcnq1ot1 maternally methylated DMR (also called IC2), and additional misexpressed imprinted genes. These results have revealed that correct insulator dose and strength at the *H19-Igf2* ICR is required for perinatal viability: strict biallelic insulation at this imprinted locus is not tolerated in development.

## CTCF is the major epigenetic organizer of the maternal allele in the soma

**Figure 3:**
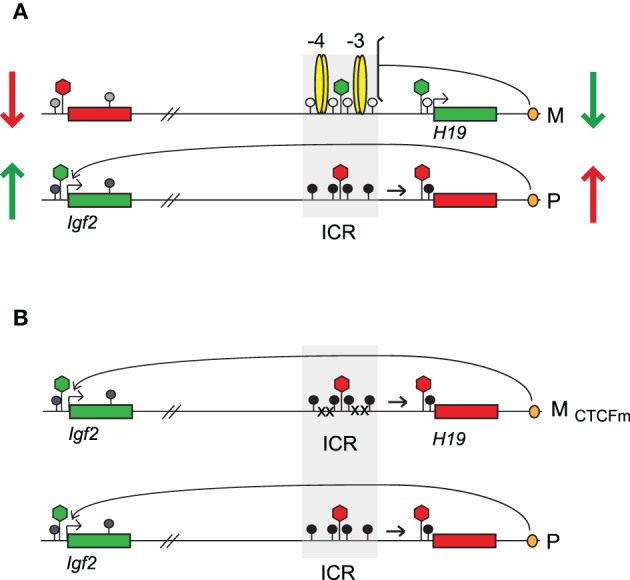
**CTCF is the major epigenetic organizer of the maternal allele in the soma. (A)** Domain-wide allele-specific epigenetic features of the *H19*/*Igf2* imprinted domain. DNA methylation is paternal allele-specific in the ICR and at the *H19* promoter. Methylation is also paternally biased at the *Igf2* promoter and DMR2 (lollipops with shades of gray). Histone covalent modificatons are polarized along the domain. Active chromatin marks (green hexagon) exist at the active gene copies and in the maternal ICR but repressive marks (red hexagon) exist in the silent gene copies and the paternal ICR. **(B)** CTCF binding in the ICR is required for domain-wide epigenetic features. The maternal chromosome that carries CTCF binding site mutations (M_CTCFm_) becomes very similar to the normal paternal chromosome in each epigenetic feature, DNA methylation and chromatin composition. Vertical arrows in **(A)** depict the changes in enrichment of active (green) and repressive (red) chromatin marks at the *Igf2* or *H19* regions that occur in response to CTCF site mutations.

CTCF is the master organizer of the maternal allele's chromatin (Figure 3). Utilizing single nucleotide polymorphisms (SNPs) between parental mouse lines and using quantitative allele-specific chromatin immunoprecipitation single nucleotide primer extension (SNuPE) assays, we measured the chromatin composition along the *H19/Igf2* imprinted domain in normal cells and cells with engineered mutations at the four ICR-CTCF binding sites. The chromatin composition showed great polarization along the *H19*/*Igf2* imprinted domain (Han et al., 2008; Singh et al., 2010a,b, 2011). Whereas the *H19* gene, promoter, and ICR were enriched in active chromatin marks, H3K4me2, H3K4me3, and H3K9ac in the maternal allele, the paternal allele of the same regions was enriched in repressive chromatin marks, such as H3K9me3 and H3K79me3. The ICR was slightly maternally biased for H3K4ac, H3K18ac, H3K36ac, H3K79ac, H4K5ac, H4K8ac, H4K12ac, and H4K91ac marks, but showed biallelic H3K27me3 enrichment. The *Igf2* promoter, DMR1 and DMR2 regions, were enriched in active marks, H3K4me2, H3K4me3, H3K9ac, H3K4ac, H3K18ac, H3K36ac, H3K79ac, H4K5ac, H4K8ac, H4K12ac, H4K91ac, H3K79me1, and H3K79me2 in the paternal allele but repressive marks, H3K27me3, H3K9me3 and repressive histone variant macroH2A1 in the maternal allele.

Abolishing CTCF binding in the *H19-Igf2* ICR in the mutant cells resulted in a complete reorganization of the allele-specific chromatin composition (Han et al., 2008). In the maternal allele CTCF site mutant cells exhibited reduced H3K9ac, H3K4me2, and H3K4me3 at the *H19* ICR, promoter, gene body and reduced H3K27me3 at the *Igf2* P2 promoter and *Igf2* DMRs. These results revealed that ICR-CTCF binding is required for recruiting the maternal allele-specific active marks, H3K9ac, H3K4me2, and H3K4me3 at the *H19* locus and the maternal allele-specific repressing mark H3K27me3 and macroH2A1 at the *Igf2* locus. In agreement with these findings, it was shown that active histone tail modifications at the *H19* promoter depend on the activity state of the promoter (Verona et al., 2008) and that CTCF directly recruits the polycomb protein Suz12 to the *Igf2* locus to catalyze H3K27 trimethylation (Li et al., 2008a). In the paternal allele H3K27me3 and macroH2A1 levels increased and became biallelic in the CTCF site-mutant cells at the *H19* promoter while paternal H3K4me2 and H3K9ac increased and became biallelic at the *Igf2* DMRs. Indeed, histone acetylation at each lysine residue increased and became biallelic in the mutant cells at the *Igf2* DMR1, P2 promoter and DMR2, where it was paternal allele-specific in normal cells (Singh et al., 2010a). These results provided evidence that in the absence of CTCF binding, the mutant maternal chromosome accumulates histone marks that normally exist in the paternal chromosome. Therefore, CTCF binding in the ICR is required for excluding repressive chromatin from the *H19* region and excluding active chromatin, such as histone acetylation from the maternal allele at the *Igf2* locus at a distance.

When we examined how CTCF binding affects the histone globular domain modifications in the *H19-Igf2* imprinted domain (Singh et al., 2010b), we found that the ICR CTCF site point mutations caused a twofold increase in the heterochromatin mark H3K79me3 at the ICR sequences. Whereas it was strongly paternal allele-specific in normal cells, H3K79me3 became biallelic in the mutant cells at the ICR and at the *H19* promoter, providing evidence that at these sequences CTCF is required for excluding H3K79me3 from the maternal allele. The ICR CTCF site point mutations also caused a twofold increase of H3K79me1 and H3K79me2 levels in the mutant cells at the *Igf2* P2 promoter and *Igf2* DMRs where these paternal allele-specific activating chromatin marks became biallelic. H3K79me1 and H3K79me2 levels were low in abundance and biallelic at the *H19* locus and H3K79me3 levels were relatively high and biallelic at the *Igf2* regions, but these features did not change in response to the CTCF site mutations, indicating that CTCF-ICR binding is not responsible in the maternal allele for including H3K79me2 at the *H19* region and H3K79me3 at the *Igf2* locus. Taken together, with regard to globular domain modifications, the ICR CTCF site mutations have caused the paternalization of the maternal allele's chromatin composition along the *H19*/*Igf2* imprinted domain by exclusion: CTCF was responsible for the maternal allele's chromatin composition by excluding H4K91ac, H3K79me1, and H3K79me2 at the *Igf2* locus and by excluding H3K79me3 at the *H19* locus from the maternal allele.

In summary, with regard to histone tail modifications, in the maternal allele CTCF binding recruited active chromatin at the *H19* locus and repressive chromatin at the *Igf2* locus, and also excluded repressive chromatin at the *H19* locus and active chromatin from the *Igf2* locus (Han et al., 2008; Singh et al., 2010a). However, CTCF did not recruit globular domain modifications to the maternal allele, rather excluded them from the maternal allele at the *Igf2* locus (Singh et al., 2010b). It will be important to find out the mechanism of how CTCF interacts with different epigenetic modifiers in achieving the maternal allele's epiphenotype.

## Control of DNA methylation at the DMR

The key to all other parental allele-specific features at the *H19-Igf2* imprinted domain is the paternal-specific methylation of the ICR, because this determines monoallelic CTCF binding, and in turn CTCF binding determines monoallelic gene expression and maintenance of the polarized epigenetic features. It is important, therefore, to review here the imprint cycle of the ICR and discuss how this cycle is related to CTCF. The methylation mark in the *H19/Igf2* ICR is erased between generations in PGCs (Hajkova et al., 2002) and is subsequently reestablished specifically in male fetal germ cells (Davis et al., 1999, 2000; Ueda et al., 2000; Kato et al., 2007). After that ICR methylation is maintained throughout spermatogenesis, fertilization, global epigenomic reprogramming in the zygote, preimplantation, and later during cell divisions in the soma (Li et al., 1993; Tucker et al., 1996; Hirasawa et al., 2008).

It is not known what initiates the paternal-specific methylation at the *H19-Igf2* DMR in the male germ line, but it depends on the *de novo* methyltransferase Dnmt3a and its cofactor, Dnmt3L (Bourc'his et al., 2001; Kato et al., 2007; Kaneda, 2011). Even though the CTCF binding sites maintain allele-specific methylation differences in the soma (see below), the same sites are not required for setting the gametic imprint in the germ line. The ICR that harbors CTCF site mutations is fully methylated in perinatal male fetal germ cells and is fully unmethylated in fetal female germ cells and ovulated oocytes (Schoenherr et al., 2003; Szabó et al., 2004). CTCF protein may affect the maintenance of unmethylated ICR in the oocyte indirectly, because CTCF-depleted oocytes exhibit increased methylation at that region (Fedoriw et al., 2004). The methylation imprinting process at the ICR in the male germ line appears to depend on two components, the ICR sequences and also the location of the ICR inside the *H19-Igf2* domain. The (ChβGI)_2_ and the (mChβGI)_2_ inserts (Figures 1C and D) attained only 11 and 32% methylation in place of the ICR in 18.5 days post-coitum (dpc) prospermatogonia, respectively, suggesting that ICR sequences are important for full methylation establishment in the male germ line (Szabó et al., 2002; Lee et al., 2010). When the ICR was introduced to other genomic locations, methylation imprint establishment did not occur in the male germ line, but paternal allele-specific methylation was acquired only later in the soma. However, when the ICR was placed downstream of the *H19* gene, it attained *de novo* methylation in the male germ line (Park et al., 2004; Tanimoto et al., 2005; Matsuzaki et al., 2009, 2010; Gebert et al., 2010). These studies suggested that the *H19-Igf2* domain's genomic location is also important for proper imprint establishment of the *H19-Igf2* ICR. It will be important to find the DNA sequences—inside and outside the ICR—that are necessary and sufficient for the mechanism of methylation imprint establishment of the ICR in prospermatogonia.

After imprint establishment the methylation of the *H19-Igf2* DMR is protected in the zygote's paternal pronucleus during the wave of zygotic reprogramming (Mayer et al., 2000; Gu et al., 2011; Iqbal et al., 2011; Wossidlo et al., 2011) by the PGC7 protein (Nakamura et al., 2007). PGC7 is proposed to protect the *H19-Igf2* DMR from 5mC oxidation by Tet3 methylcytosine oxidase in a H3K9me2-dependent manner, similarly to how PGC7 protects the female pronucleus (Nakamura et al., 2012). H3K9me2 association at this locus is inherited from the sperm and may be sufficient to attract tight PGC7 binding, which in turn is expected to reduce Tet3 affinity to these regions (Nakamura et al., 2012). The repressor protein MBD3 is slightly biased toward the paternal allele of the ICR in ES cells and, according to MBD3 knockdown experiments, contributes to protecting CpG methylation of the paternal allele of the *H19-Igf2* DMR during preimplantation development (Reese et al., 2007). Genetic studies revealed that two additional proteins protect the ICR methylation during early development. Zfp57 transcription factor protects the ICR in ES cells (Zuo et al., 2012) and Trim28 (also known as KAP1) protects it in the embryo (Messerschmidt et al., 2012). Trim28 binds to the ICR in midgestation stage embryos (Messerschmidt et al., 2012). Both Zfp57 and Trim28 are associated with the methylated paternal allele of the ICR In ES cells (Quenneville et al., 2011). Zfp57-Trim28-Setdb1 triple occupied ChIP-sequencing peaks defined a consensus hexanucleotide sequence, TGC^m^CGC where the CpG site is methylated (Quenneville et al., 2011). This consensus is present at each DMR, including the *H19-Igf2* ICR.

In somatic organs, the maternal allele's epigenetic profile at the *H19-Igf2* domain depends on CTCF binding in the ICR. CTCF binding is responsible for protecting the maternal allele from DNA methylation (Figure 3). Maternal inheritance of mutations in the CTCF binding sites resulted in highly elevated CpG methylation levels in somatic organs at the ICR (Pant et al., 2003; Schoenherr et al., 2003; Szabó et al., 2004), as well as the *H19* promoter, and *H19* gene body and even at the *Igf2* DMR1 and DMR2 sequences at ~90-kb distance (Kurukuti et al., 2006; Han et al., 2008).

**Figure 4:**
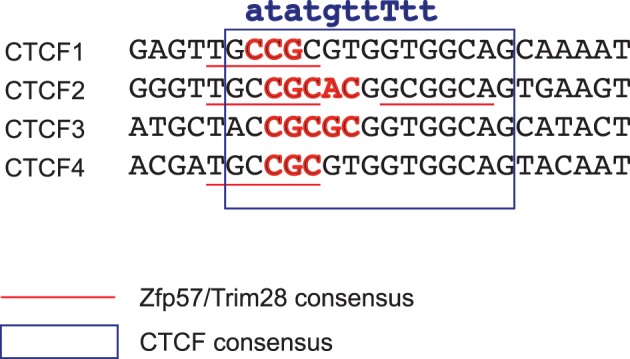
**Overlapping binding sites in the ICR for CTCF and Zfp57-Trim28-Setdb1 repressor complex explain their antagonistic roles at the ICR.** The four CTCF binding sites (inside the blue rectangle) of the ICR are shown with the intertwined consensus sequences defined by Zfp57-Trim28-Setdb1 (underlined in red). The nucleotides that were mutated by point mutations (Engel et al., 2004) that specifically destroy Zfp57-Trim28-Setdb1 consensus sequence are in red. Point mutations that abolish CTCF binding and also destroy Zfp57-Trim28-Setdb1 consensus sites are written in the top line.

It is interesting to note that the Zfp57-Trim28-Setdb1 consensus sites overlap with three CTCF binding motifs in the ICR (Figure 4). At these sites the maternal allele has robust *in vivo* CTCF footprints in MEF. However, in MEFs no clear DNAseI footprints are discernable in the paternal allele (Szabó et al., 2000). Zfp57-Trim28 binding may only take place in the ICR at earlier time points, before the time of MEF derivation. Incidentally, the Zfp57-Trim28-Setdb1 consensus sites have been mutated in the *H19-Igf2* ICR (well before the consensus site was discovered) at the endogenous locus and in integrated transgenes (Engel et al., 2004; Matsuzaki et al., 2010). These mutations destroyed the Zfp57-Trim28-Setdb1 consensus sites such way that CTCF binding was not affected (Figure 4). As a result, methylation was reduced and insulator activity was gained in the mutant paternal ICR, likely because the reduced DNA methylation allowed CTCF binding. Zfp57-Trim28 may protect the ICR from demethylation by attracting repressing epigenetic modifiers and DNMTs to the target sequences and by facilitating heterochromatinization and DNA remethylation (Quenneville et al., 2011; Zuo et al., 2012), although this function may be redundant, because the Zfp57 null mutant midgestation embryos did not exhibit reduced ICR DNA methylation (Li et al., 2008b). It is interesting that Zfp57-Trim28-mediated protection of DNA methylation is required in the *H19-Igf2* ICR only when CTCF binding sites are present. When the CTCF consensus was destroyed together with the Zfp57-Trim28-Setdb1 consensus (Figure 4), DNA methylation maintenance was not affected (Szabó et al., 2004). Zfp57-Trim28's role at the ICR, therefore, is specific to preventing CTCF binding in the paternal allele by maintaining DNA methylation. One extension of this idea is that CTCF may protect the maternal allele from DNA methylation by preventing Zfp57-Trim28-Setdb1 binding. Therefore, the antagonistic roles (Engel et al., 2004) of the composite ICR CTCF sites are the following: to maintain the methylation-free status of the maternal chromosome through CTCF binding and to maintain DNA methylation in the paternal chromosome through Zfp57-Trim28-Setdb1 binding.

## CTCF-dependent chromatin bias delays *de novo* methylation of the maternal ICR allele in male germ cells

**Figure 5:**
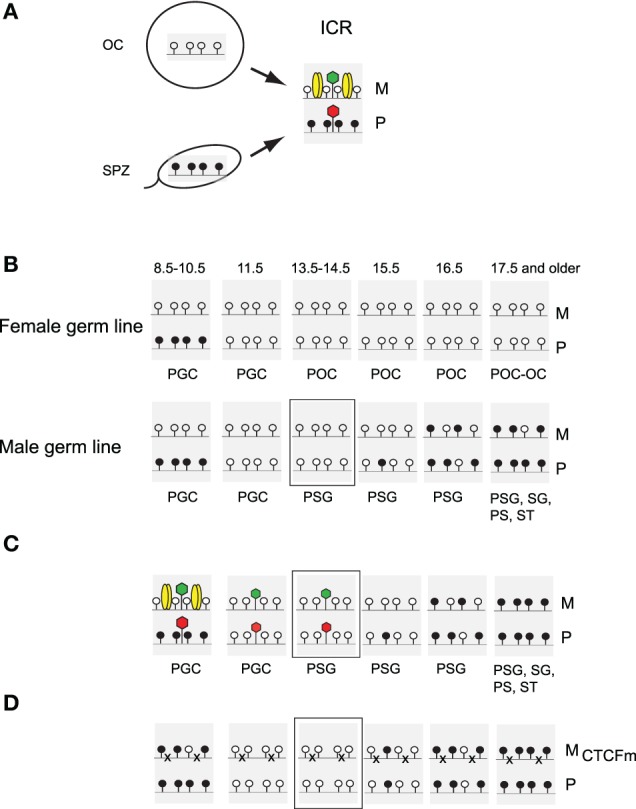
**CTCF binding delays *de novo* methylation of the maternal allele in male germ cells. (A)** Differential methylation of the ICR is inherited from the gametes: methylation of the paternal allele (P) from spermatozoa (SPZ) and unmethylation of the maternal allele (M) from oocytes (OC). This primary methylation difference determines CTCF binding and chromatin composition in the soma and likely also in primordial germ cells (PGC), which exhibit imprinted *H19* and *Igf2* expression. Active or repressive chromatin (green or red hexagon) is present at respective alleles of the ICR. **(B)** Fate of the imprint in the female and male germ lines. Methylation status of the ICR is depicted in the primordial germ cells (PGC), primary oocytes (POC) and in prospermatogonia (PSG), spermatogonia (SG) pachytene spermatocytes (PS) and round spermatids (ST) with gestational stages in dpc. The developmental stage that appears epigenetically different without DNA methylation is marked with a rectangle. **(C)** Imprint establishment of the ICR in the normal male germ line. Expected CTCF binding and chromatin composition is depicted in primordial germ cells (PGC). Observed chromatin bias is depicted in prospermatogonia (PSG). Chromatin bias is observed in the normal ICR between the parental alleles in the absence of CpG methylation at 13.5–14.5 dpc. **(D)** Functional CTCF sites are required for chromatin bias and delayed methylation of the maternally inherited ICR allele. Maternal inheritance of the CTCF binding site mutations abolishes CTCF binding in the maternal allele in PGCs. No chromatin bias is observed between parental alleles at 13.5–14.5 dpc and the maternal allele's methylation is not delayed at 15.5–17.5 dpc.

The process of methylation imprint erasure at the ICR is complete in PGCs by 13.5 dpc (Figure 5). Consequently, male fetal germ cells undergo *de novo* methylation at the ICR during fetal development, whereas female germ cells remain unmethylated till the end of oocyte maturation. It was noticed by several laboratories that the two ICR alleles are different in male germ cells with respect to the speed of *de novo* methylation. Methylation of the paternally inherited ICR allele precedes the maternally inherited allele (Davis et al., 1999, 2000; Ueda et al., 2000; Kato et al., 2007), implying that the two alleles are distinguished by an epigenetic mark, other than DNA methylation in 13.5 dpc prospermatogonia. We hypothesized that the chromatin composition may constitute this transient epigenetic memory and this in turn depends on maternal-allele-specific binding of CTCF in PGCs. In order to test our hypothesis we isolated fetal germ cells from mice that carry SNPs at the ICR to distinguish the parental chromosomes. Using allele-specific ChIP-SNuPE and real-time reverse-transcription PCR assays we found that CTCF was slightly biased toward the maternal allele, but it had a very low level of enrichment at 13.5 dpc at the ICR, suggesting that CTCF is almost completely removed from the ICR in germ cells before midgestation. The repressive histone mark, H3K9me3, was slightly biased toward the paternal allele at the ICR but its enrichment level was very low whereas the active mark, H3K4me2 was more abundant and it was slightly biased toward the maternal allele in prospermatogonia at 13.5 and 15.5 dpc. The level of H3K4me2 allelic bias was similar to the methylation bias between alleles (10–15%). When the maternal allele carried the CTCF site mutations in prospermatogonia, the chromatin bias was no longer observed at the ICR, suggesting that chromatin composition of the ICR depends on maternal-allele specific CTCF binding in PGCs, just like it does in somatic cells (Han et al., 2008; Singh et al., 2010a,b). The methylation bias was also absent between the parental alleles in the mutant prospermatogonia. These findings are consistent with the explanation that CTCF binding in PGCs is responsible for setting up a chromatin bias in PGCs, and that this chromatin is not fully erased in prospermatogonia before *de novo* methylation commences. Therefore, CTCF-dependent chromatin bias may influence the rate of DNA methylation in the parental alleles. We concluded that it is the H3K4me2 histone mark that most likely constitutes the epigenetic memory of the mother in prospermatogonia at 13.5–14.5 dpc and delays *de novo* CpG methylation in the maternal ICR allele. Indeed, removal of H3K4me2 by H3K4 demethylase KDM1B is required at least at certain maternal DMRs for the establishment of methylation imprints in oocytes (Ciccone et al., 2009). It is known that certain maternal DMRs exhibit delayed *de novo* methylation in the paternally inherited allele (Hiura et al., 2006). It will be interesting to find out using genetic analyses whether CTCF or other transcription factor provides transient epigenetic memory for those alleles.

In summary, CTCF plays complex roles at the *H19-Igf2* ICR. All of these roles may appear at first to depend on its major role at the domain, which is enhancer blocking. However, CTCF also protects the ICR from DNA methylation in the maternal allele and also sets up the maternal allele's chromatin composition in the soma and to some extent in PGCs. These functions at a single locus illuminate the versatility of CTCF in organizing gene expression and also in structuring the genome. It will be important to carry out similar genetic experiment by precisely inactivating the binding sites using point mutations to understand whether CTCF organizes local and domain-wide chromatin composition and/or maintains the unmethylated state at other loci in the genome, especially those that where insulator function has been shown (Herold et al., 2012). At least at one other locus, at the β-globin cluster 3′HS1, CTCF binding was shown to be required for recruiting active chromatin mark H3K9ac and repelling the repressing marks H3K9/27me3 (Splinter et al., 2006). We will be very curious to see whether CTCF binding sites in the Xist/Tsix RS14 region (Spencer et al., 2011) regulate the choice of X chromosome for inactivation by orchestrating local or domain-wide chromatin composition. Interestingly, mutations in the corresponding human sites either increase or decrease CTCF binding affinity and also reciprocally affect X inactivation skewing (Pugacheva et al., 2005). It will be especially critical to find out whether CTCF carries out its chromatin organizing activities parental allele-specifically at other imprinted domains and if proper CTCF binding at those DMRs is essential for development. We expect that this will be true at least at the *Dlk1-Gtl2* imprinted domain, because CTCF binding is allele-specific in a strategically important location at the *Gtl2* promoter (Lin et al., 2011) and because of the known lethality phenotypes associated with the misregulation of allele-specific expression at this imprinted domain (Lin et al., 2003; Wu et al., 2006; Takahashi et al., 2009, 2010).

### Conflict of interest statement

The authors declare that the research was conducted in the absence of any commercial or financial relationships that could be construed as a potential conflict of interest.
